# CNN-stylized dual-path CNN–Transformer for fetal head and pubic symphysis segmentation in intrapartum ultrasound

**DOI:** 10.3389/fphys.2026.1802136

**Published:** 2026-07-01

**Authors:** Zhensen Chen, Wenbin Luo, Yaosheng Lu

**Affiliations:** 1 School of Computer Engineering, Jimei University, Xiamen, China; 2 Department of Radiology, The Second Affiliated Hospital of Xiamen Medical College, Xiamen, Fujian, China; 3 College of Information Science and Technology, Jinan University, Guangzhou, China

**Keywords:** boundary attention, CNN-stylized Transformer, fetal head and pubic symphysis segmentation, intrapartum ultrasound, medical image processing

## Abstract

In intrapartum ultrasound (IU) imaging, reliable segmentation of the fetal head (FH) and the pubic symphysis (PS) is a prerequisite for automated quantification of the angle of progression (AoP), a clinically meaningful indicator for predicting delivery outcomes and mitigating maternal–neonatal complications. However, existing CNN–Transformer hybrid models often suffer from unstable attention learning under limited annotated medical data, which can lead to attention collapse. In addition, they tend to underexploit boundary cues in IU images, where contours are frequently degraded by noise and artifacts. To address these issues, we propose a CNN-stylized dualpath CNN–Transformer framework tailored for IU segmentation. The encoder consists of a CNN branch and a CNN-stylized Transformer branch to balance local detail representation and long-range dependency modeling while improving the robustness of attention learning. A Transformer-to-CNN fusion module is further introduced to enhance inter-branch interaction and feature complementarity. In the decoder, a Reverse-Additive Boundary Refinement module explicitly models the foreground–background transition region, progressively refining boundary representations. Extensive experiments on three datasets demonstrate that the proposed method consistently achieves superior overall segmentation accuracy and improved boundary quality compared with state-of-the-art approaches.

## 1 Introduction

**Figure 1:**
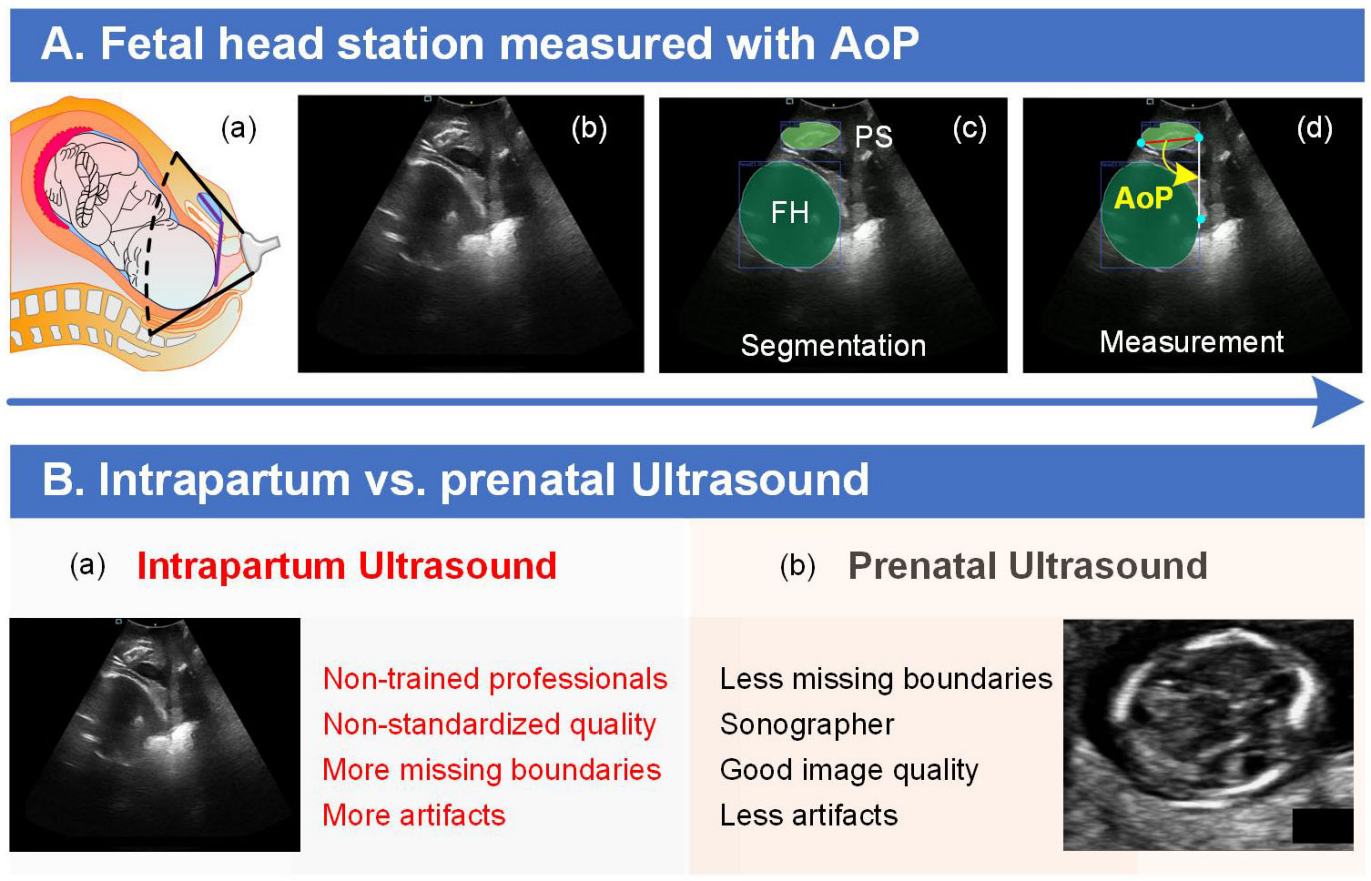
**(A)** The figure depicts the process of measuring the angle of progression (AoP) utilizing transperineal ultrasound. The AoP is defined as the angle formed between a straight line drawn along the longitudinal axis of the pubis symphysis (PS) and a line extending from the lowermost point of the PS to the leading edge of the fetal head (FH). (a) Schematic representation illustrating the computation of AoP. (b) An intrapartum ultrasound image containing PS and FH. (c) Segmentation results of PS and HF. (d) The calculation of AoP accomplished through elliptic function fitting. **(B)** Shows the difference between prenatal ultrasound and intrapartum ultrasound. (a) Intrapartum ultrasound. (b) Prenatal ultrasound.

Monitoring labor progression is foundational to safe perinatal care: it supports timely clinical decisions, facilitates appropriate intervention, and ultimately reduces risks for both mother and fetus. Among the indicators used to judge how labor is unfolding, the position of the fetal head plays a particularly influential role Sandall et al. (2018). In many settings, this information is still obtained through vaginal examinations, as recommended by the WHO Malvasi et al. (2014). Yet conventional digital assessment is well known to be subjective and difficult to reproduce across examiners, and its invasive nature can cause substantial discomfort. These limitations have helped drive the clinical adoption of intrapartum ultrasound (IU) as a more reliable alternative Ramphul et al. (2014); Bellussi et al. (2017). In line with this shift, both ISUOG and WAPM highlight IU as an objective and precise tool for assessing fetal position Ghi et al. (2018); Rizzo et al. (2022). Within this framework, the angle of progression (AoP) has become a prominent quantitative marker of fetal head descent Ghi et al. (2018). Clinically, an AoP ≥ 120° is commonly interpreted as supportive of vaginal delivery, whereas AoP *<* 120° is more suggestive of cesarean section Dall’Asta et al. (2019). Crucially, AoP is not a direct readout of the image: it depends on accurate identification of two anatomical structures—the fetal head and the pubic symphysis—as illustrated in **Figure 1A**.

Despite its value, AoP assessment remains largely manual in routine practice. Clinicians typically estimate it by visually integrating image layout, echogenic patterns, and perceived boundary cues—an approach that is time-consuming and vulnerable to inter-observer variability Rizzo et al. (2022); Ling et al. (2023); Kuang et al. (2022); Xing et al. (2024). The challenge is amplified by the nature of IU itself. Unlike antenatal ultrasound, which is often acquired under controlled conditions by trained sonographers, IU is performed during active labor—frequently by obstetricians or nurses and under urgent clinical constraints. Motion, physiological changes, and probe–tissue dynamics introduce artifacts and noise that can obscure or blur the margins of the fetal head (FH) and the pubic symphysis (PS), as shown in **Figure 1B**. Consequently, robust FH/PS segmentation becomes the bottleneck for dependable automated AoP quantification. Recognizing the need for standardized evaluation and benchmarking, we organized the first IU-based FH–PS segmentation challenge at MICCAI 2023 Bai et al. (2024).

Technically, segmentation in this setting sits at the intersection of two modeling demands: capturing fine-grained boundary details while also leveraging broader contextual cues to resolve ambiguity. Encoder–decoder architectures such as UNet Ronneberger et al. (2015) have become the workhorse of medical image segmentation, with convolutional neural networks (CNNs) offering strong local representation capacity. However, CNNs can struggle to model long-range dependencies that are often critical for consistent delineation Wang et al. (2018). Transformers provide a complementary mechanism by directly modeling global interactions, but they can be less responsive to subtle local structures and typically require larger datasets to train reliably—an assumption that is difficult to satisfy in medical imaging.

Hybrid CNN–Transformer designs attempt to reconcile these strengths, enabling joint modeling of local and global information Qiao et al. (2024); Tang et al. (2024); Li et al. (2024a); Mu et al. (2024). Yet, in data-limited regimes, many such hybrids exhibit a recurring failure mode: attention collapse, in which attention maps degenerate toward near-uniform patterns, diminishing the effective role of the Transformer component and implicitly shifting learning back toward the CNN pathway Wang et al. (2024); Lin et al. (2023a); Qi et al. (2024). For IU images, this instability compounds an already difficult boundary problem. FH and PS contours are frequently low-contrast and disrupted by artifacts, and purely edge-driven remedies—such as Sobel-based enhancements or directional field modeling—may sharpen contours at the cost of ignoring informative background context Lin et al. (2023b); Di et al. (2022). Complementary strategies such as boundary-aware augmentation can further encourage models to attend to the most fragile transition regions, providing an additional route to improving segmentation fidelity at clinically relevant edges.

Therefore, we are currently faced with the following three problems that need to be solved:

### 1.1 How to effectively capture local features and long-range dependencies

A persistent challenge in medical image segmentation is to represent fine-grained local structure (e.g., edges and textures) while simultaneously modeling long-range contextual relationships that disambiguate anatomies and enforce global consistency. In practice, many existing architectures lean toward one side of this spectrum, which limits their ability to exploit local–global complementarity within a single unified representation. For example, nnFormer Zhou et al. (2021b) adopts a Transformer-only encoder. Although this design is well suited for capturing global interactions, it can be less data-efficient for learning localized cues, especially when subtle boundaries or small structures dominate the segmentation target. Conversely, TransUNet Chen et al. (2021) primarily relies on a CNN encoder and introduces Transformer blocks only at the encoder output to inject global context; this late-stage integration can restrict the model’s capacity to propagate long-range dependencies throughout the hierarchy of features. Similarly, SwinUnet Cao et al. (2022), as a fully Transformer-based framework, may not consistently match CNN-style inductive biases in extracting low-level spatial details, potentially weakening performance when boundary fidelity is critical.

### 1.2 How to solve the Transformer attention collapse

A well-recognized weakness of CNN–Transformer hybrids in medical imaging is the tendency of the Transformer component to become ineffective as network depth increases. In particular, deeper representations are more susceptible to attention collapse, where attention distributions degenerate and lose discriminative structure Zhou et al. (2021a). This phenomenon is closely related to the data regime: most medical segmentation benchmarks are relatively small compared with natural-image corpora, making it difficult for Transformers to reliably learn stable attention patterns and, consequently, to exploit long-range dependencies as intended Zhang et al. (2023). In na¨ıve hybrid designs the training dynamics often bias the model toward the CNN pathway, while the Transformer branch contributes marginally—effectively reducing the hybrid to a CNN-dominated system and undermining global-context modeling. Moreover, Previous work, such as ConvFormer, has experimentally shown that introducing CNN-style inductive bias into Transformer structures can improve attention stability and alleviate attention-collapse behavior in medical image segmentation Lin et al. (2023a). These observations suggest that mitigating attention collapse requires not only architectural combination, but also mechanisms that stabilize attention learning and encourage meaningful cross-branch utilization across depths Zhou et al. (2021a); Zhang et al. (2023); Lin et al. (2023a).

### 1.3 How to effectively combine local features with long-range dependencies

A common strategy for integrating CNN-derived locality with Transformer-based global reasoning is to couple the two modules in a relatively direct manner. One line of work uses CNNs as front-end feature extractors and then passes the resulting representations into Transformer blocks to introduce long-range interaction modeling Chen et al. (2021); Xie et al. (2021). Another line of work constructs hybrid representations by aggregating the outputs of CNN and Transformer components—often through channel-wise concatenation—followed by subsequent fusion to form a combined feature space Chen et al. (2024a); Lee et al. (2022). Although these designs can yield measurable gains, such coarse coupling is not always sufficient to exploit the true complementarity between local detail encoding and long-range dependency modeling. In practice, limited cross-component communication, weak alignment between feature hierarchies, or overly shallow fusion can prevent the network from jointly optimizing locality and global context, leading to suboptimal synergy in the final segmentation representation.

To address the above challenges, we develop a new network for foreground–background segmentation in IU images. Different from recent CNN–Transformer hybrid segmentation networks that commonly introduce Transformer blocks after CNN feature extraction or fuse CNN and Transformer representations through direct concatenation, the proposed framework is designed around a CNN-stylized dual-path architecture tailored for intrapartum ultrasound segmentation. The Transformer branch is reformulated using convolutional projections, pooling-based spatial reduction, and 2D topology-preserving feature processing, thereby incorporating CNN-like inductive bias to improve attention stability under limited annotated medical data. In addition, the proposed CBFE module performs explicit cross-branch interaction by using Transformer-derived contextual cues to adaptively recalibrate CNN features from both channel and spatial perspectives, rather than relying on coarse feature aggregation. Moreover, unlike conventional boundary-aware methods that mainly depend on edge maps or auxiliary boundary supervision, the proposed RABR module uses reverse-additive attention to mine boundary-relevant cues from regions that are likely to be suppressed as background and progressively refines ambiguous foreground–background transitions through a residual refinement pathway. These designs jointly distinguish our method from existing hybrid and boundary-aware segmentation architectures and make it particularly suitable for FH/PS segmentation in noisy and artifact-prone intrapartum ultrasound images.

The main contributions of this paper are as follows:

- We introduce a new CNN–Transformer hybrid framework for FH and PS segmentation in IU, equipped with a CBFE module to promote explicit feature interaction and fusion between the convolutional and attention-based pathways.
- We design a CNN-style Transformer branch injected with a CNN-like inductive bias to stabilize attention learning on limited medical data, thereby mitigating attention collapse and improving convergence performance.
- We develop a RABR module, which uses reverse additive attention to emphasize easily missed cues in both foreground and background and employs a residual refinement scheme to progressively enhance fine structural details.

## 2 Proposed method

**Figure 2:**
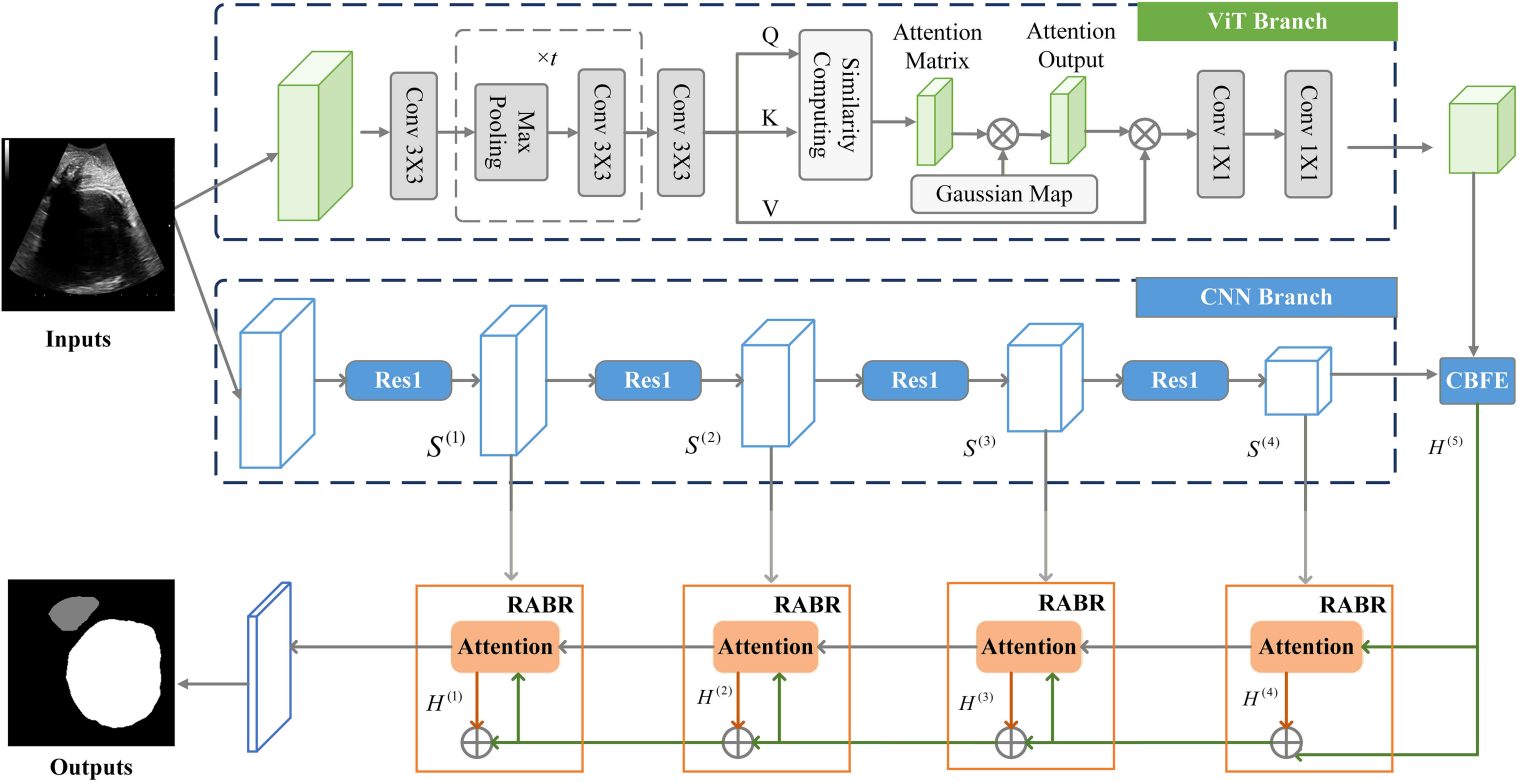
The proposed network is organized around a decoder that couples a CNN branch with a CNNstylized Transformer branch, and is further equipped with a feature interaction module and a boundary refinement module.

The proposed architecture adopts a two-stream decoder composed of a CNN pathway and a CNN-stylized Transformer pathway. This coupling is designed to retain high-frequency local structures (e.g., subtle edges) while simultaneously capturing long-range contextual dependencies. By injecting CNN-like inductive biases into the Transformer stream, the attention mechanism remains more trainable in data-scarce settings, thereby reducing susceptibility to attention collapse. To reconcile and exploit the complementary cues from both pathways, we incorporate a CBFE module, which enforces explicit cross-stream communication and produces a globally informed fused representation. In addition, we propose a RABR module to restore boundary evidence that is often attenuated in both foreground and background areas. RABR combines reverse additive attention with residual, stage-wise refinement to iteratively enhance ambiguous transitions and sharpen blurred contours. The overall network is depicted in **Figure 2**.

### 2.1 Dual-path parallel decoder

#### 2.1.1 CNN branch

The CNN branch of the dual-path encoder is designed to capture local features, utilizing Res2Net as the backbone architecture. It consists of four residual blocks, and the generated features are denoted as *C_i_*.

#### 2.1.2 CNN-stylized transformer branch

To stabilize attention learning under data-scarce medical settings and to speed up optimization, we construct a CNN-stylized Transformer branch in which tokenization and self-attention are implemented in a fully 2D, convolution-friendly manner. Unlike a standard Vision Transformer (ViT) pipeline that relies on patch tokenization and explicit positional embeddings, our branch preserves spatial topology throughout the computation, thereby avoiding additional positional encoding while retaining strong locality priors.

Given an input feature map *X*, we first apply a 3 × 3 convolution to extract low-level local patterns. Then, instead of forming patch tokens, we downsample the feature map using a repeated 2 × 2 max-pooling operator, each followed by a 3 × 3 convolution, until the resolution matches a target patch size *P* (analogous to the patch size in ViT). The number of downsampling stages *r* is computed as Equation 1.

(1) $$r=\log_{2}P.$$

After *r* stages, the pooled representation becomes Equation 2.

(2) $$X_{r}\in {\mathbb{R}}^{d\times \frac{H}{2^{r}}\times \frac{W}{2^{r}}},$$

where *d* denotes the embedding dimension of the branch.

To emulate the *Q*-*K*-*V* projections of ViT while keeping a CNN-like structure, we generate query, key, and value features using three parallel 3 × 3 convolutions. For a spatial location (*u, v*) on *X_r_*, the query and key vectors are computed by local convolutional projections, as defined in Equation 3.

(3) $$\begin{array}{lll} q_{u,v} & =\overset{1}{\underset{a=-1}{\sum }}\overset{1}{\underset{b=-1}{\sum }}W^{\left(q\right)}*a,b,x*u+a,v+b,\;k_{u,v} & =\overset{1}{\underset{a=-1}{\sum }}\overset{1}{\underset{b=-1}{\sum }}W^{\left(k\right)}*a,b,x*u+a,v+b, \end{array}$$

where $W^{\left(q\right)}$ and $W^{\left(k\right)}$ are learnable convolution kernels, and $x_{u,v}\in {\mathbb{R}}^{d}$ is the feature vector at (*u, v*).

We then form an attention-like affinity between (*u, v*) and any location (*m, n*) using cosine similarity, as defined in Equation 4.

(4) $$s^{u,v}*m,n=\frac{\langle q*u,v,,k_{m,n}\rangle }{|q_{u,v}|*2|k*m,n{|}_{2}+\epsilon },$$

where ⟨·, ·⟩ is the dot product, $|\cdot {|}_{2}$ is the *ℓ*_2_ norm, and *ϵ* is a small constant.

To adaptively expand the effective receptive field, we introduce a Gaussian distance prior that modulates affinities based on spatial distance, as defined in Equation 5.

(5) $$g_{m,n}^{u,v}=\text{exp }(-\frac{{\left(u-m\right)}^{2}{\left(\frac{2^{r}}{H}\right)}^{2}+{\left(v-n\right)}^{2}{\left(\frac{2^{r}}{W}\right)}^{2}}{2{\left(\lambda ,\mu \right)}^{2}}),$$

where *λ* is a preset hyper-parameter controlling the expansion trend, and *µ* is a learnable scale factor. As *λ* increases (or as *µ* is learned larger), the kernel gradually approaches a more global range.

We combine the similarity map and the distance prior to obtain adaptive weights, as defined in Equation 6.

(6) $$a^{u,v}*m,n=\text{Softmax }*m,n(s^{u,v}*m,n\cdot g^{u,v}*m,n),$$

so that each location (*u, v*) receives an adaptive aggregation kernel $a^{u,v}$.

The value vectors are generated in the same convolutional manner, as defined in Equation 7.

(7) $$v_{u,v}=\overset{1}{\underset{a=-1}{\sum }}\overset{1}{\underset{b=-1}{\sum }}W^{\left(v\right)}*a,b,x*u+a,v+b,$$

and the long-range aggregated output at (*u, v*) is computed as defined in Equation 8.

(8) $$y_{u,v}=\underset{m,n}{\sum }a^{u,v}*m,n,v*m,n.$$

Finally, we apply a 1 × 1 convolution to project the aggregated features into the desired channel space.

To strengthen long-range dependency modeling while maintaining computational efficiency, we instantiate a single CNN-stylized Transformer branch with a fixed downsampling depth of *r* = 4, corresponding to an effective patch size of *P* = 16. This setting provides a practical balance between contextual modeling and spatial-detail preservation. When the downsampling depth is too small, the feature map remains relatively high-resolution, which increases the computational cost of attention-like aggregation and limits the effective receptive-field expansion. In contrast, an excessively large downsampling depth produces overly coarse representations, which may suppress fine boundary information that is critical for delineating the fetal head and pubic symphysis. Therefore, *r* = 4 was selected to obtain a compact yet sufficiently informative representation, allowing the Transformer branch to capture long-range dependencies while preserving boundary-relevant spatial cues. To support efficient inference for real-time intrapartum ultrasound analysis and edge-device deployment, the computational complexity is formulated in Equation 9.

(9) $$F_{\text{tr}}=\phi_{1\times 1}\;(Y_{4}).$$

Because this branch is built from pooling and convolutions, it remains computationally efficient and, importantly, inherits CNN-like inductive biases that improve training stability—helping to alleviate attention collapse and accelerating convergence in limited medical-data regimes.

### 2.2 Cross-branch feature exchange block

Because the decoder contains two complementary streams—one emphasizing local convolutional cues and the other encoding long-range context—performance depends on how effectively these representations are coupled. Notably, our CNN-stylized Transformer stream preserves 2D spatial topology and can be treated as a context-rich convolutional pathway. Motivated by this observation, we design a CBFE block to inject global contextual dependencies from the Transformer stream into the convolutional stream, thereby recalibrating and strengthening local features. Let $F^{c}\in {\mathbb{R}}^{C\times H\times W}$ denote the feature map from the CNN stream and $F^{t}\in {\mathbb{R}}^{C\times H\times W}$ the feature map from the Transformer stream (after resolution alignment). CBFE generates a joint gating map by combining channel-wise and spatial-wise modulation.

We first compute a channel descriptor via global average pooling, as defined in Equation 10.

(10) $$z=\text{GAP }(F^{t})\in {\mathbb{R}}^{C}.$$

The descriptor is then passed through two pointwise convolutions (implemented as 1 × 1 convolutions), serving as lightweight channel context aggregators, as defined in Equation 11.

(11) $$g_{c}=\psi_{2}(\psi_{1}(z))\in {\mathbb{R}}^{C},$$

where *ψ*_1_(·) and *ψ*_2_(·) denote learnable 1 × 1 projections.

To capture spatial cues at different receptive-field ranges, CBFE employs two depthwise separable convolutions with kernel sizes 5 × 5 and 7 × 7, as defined in Equation 12:

(12) $$g_{s}=\delta_{5}(F^{t})⊕\delta_{7}(F^{t})\in {\mathbb{R}}^{C\times H\times W},$$

where $\delta_{k}(\cdot )$ denotes a depthwise separable convolution with kernel size *k*×*k*, and ⊕ indicates element-wise summation (or you may use concatenation followed by a 1 × 1 projection).

Finally, we combine channel and spatial modulators to form an attention-like weight map, as defined in Equation 13.

(13) $$A=\sigma (\text{Broadcast }(g_{c})⊙g_{s}),$$

where *σ*(·) is the Sigmoid function, ⊙ denotes element-wise multiplication, and Broadcast(·) expands *g_c_*to *C* × *H* × *W*. The refined CNN-stream feature is obtained as defined in Equation 14.

(14) $${\tilde{F}}^{c}=F^{c}⊙A+F^{c}.$$

**Figure 3:**
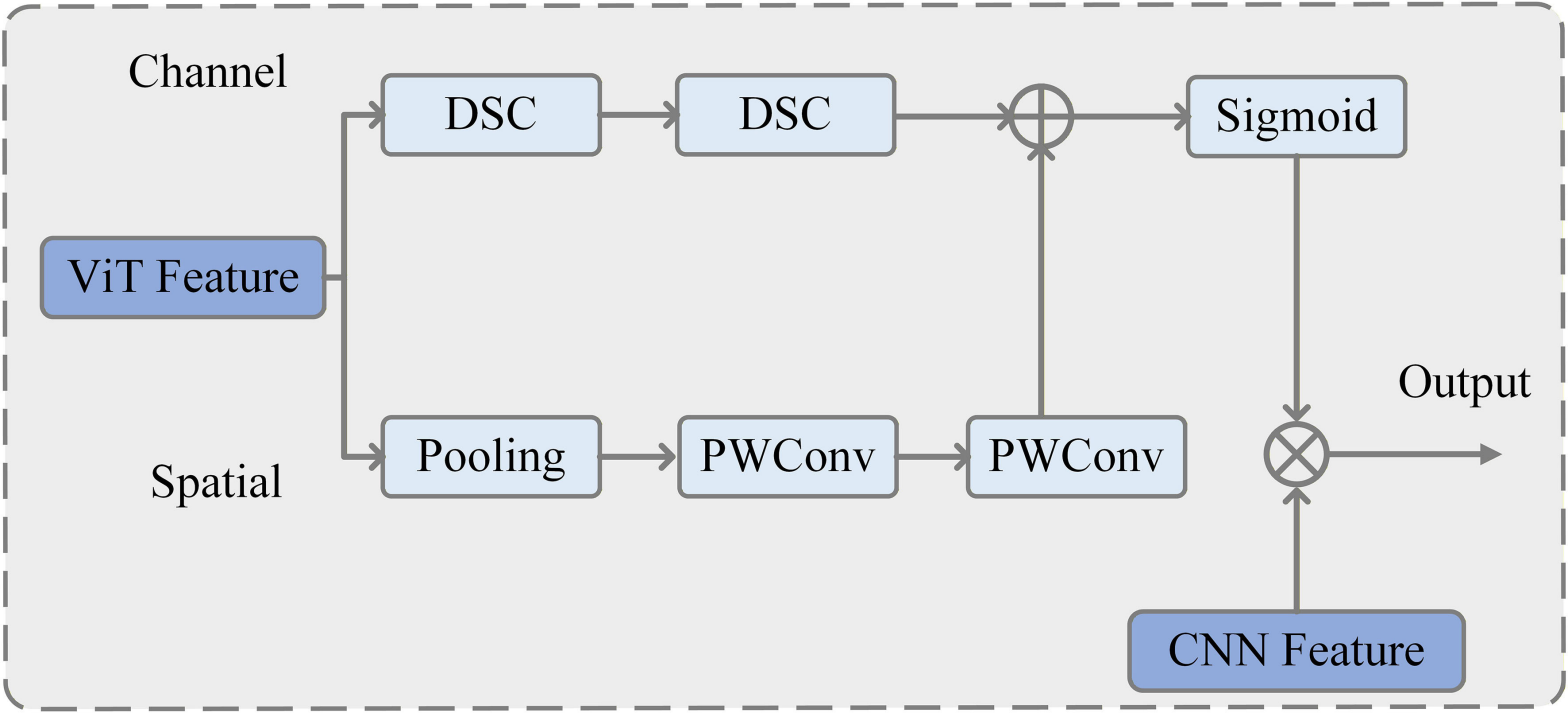
The CBFE module aggregates representations from the CNN stream and the CNN style Transformer stream, performing joint fusion across spatial layouts and channel-wise semantics to form a more holistic descriptor.

This design enables the CNN stream to be adaptively reweighted by Transformer-derived global context while preserving its local-detail fidelity through the residual pathway. The CBFE block is illustrated in **Figure 3**.

### 2.3 Reverse-additive boundary refinement

**Figure 4:**
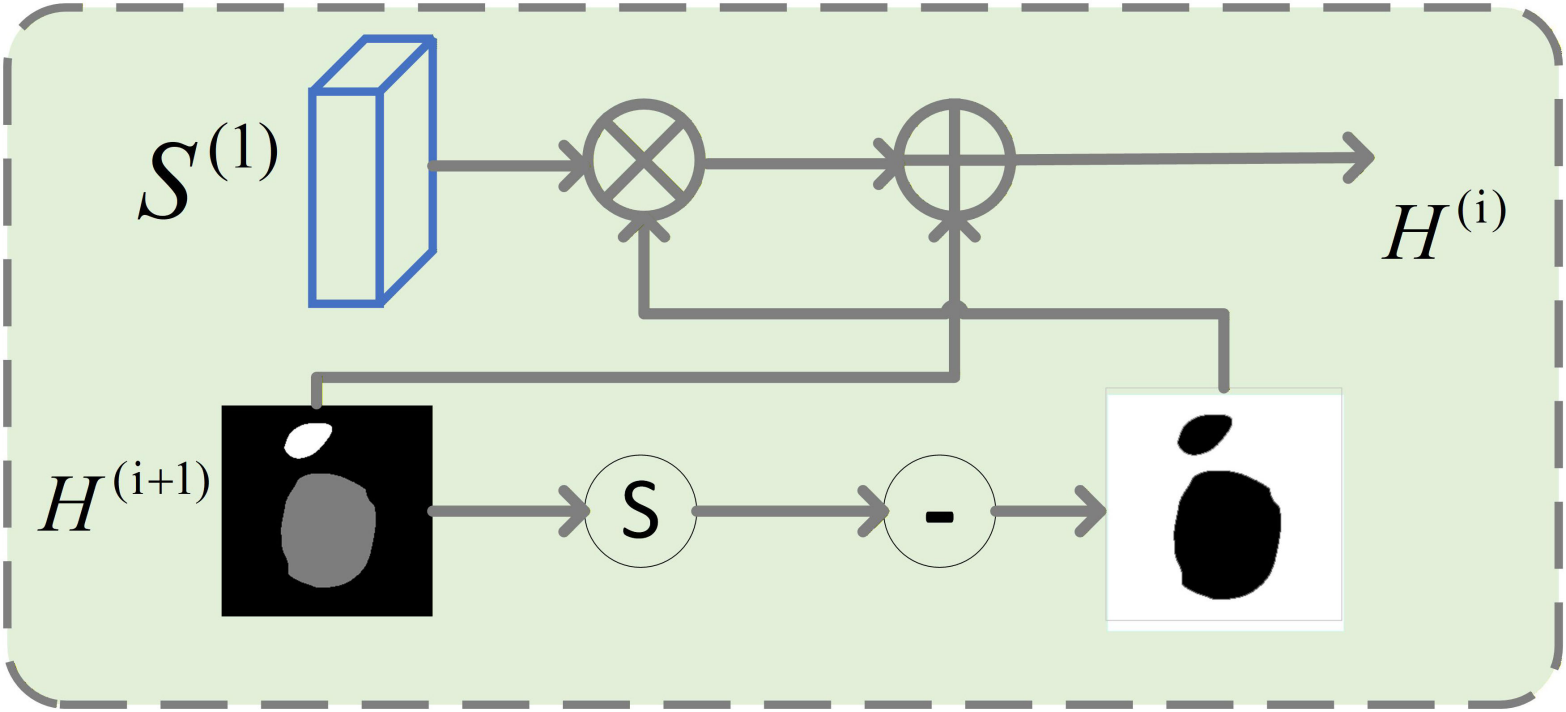
The RABR module leverages the encoder’s side outputs as auxiliary guidance, mining discriminative cues that are often buried in background responses, and then progressively enhances these cues via residual refinement to yield sharper structural details.

To inject high-resolution structural cues from the CNN encoder into the hybrid decoder representation, we design a RABR module, the boundary-attention unit is illustrated in the **Figure 4**. RABR is driven by the observation that boundary errors frequently arise when parts of the foreground are mistakenly suppressed into the background. Therefore, we explicitly mine such missing boundary evidence by constructing a reverse additive attention gate from the decoder’s coarse/global prediction and using it to reweight encoder-side details. A residual refinement pathway is then used to progressively sharpen ambiguous transitions.

RABR takes two sources of information: (i) a decoder feature $H^{\left(i+1\right)}$ propagated from the previous stage (originating from the CBFE output at the top level), and (ii) a lateral high-resolution feature *S*^(^*^i^*^)^ from the *i*-th encoder stage of the CNN branch.

We first project the decoder feature into a soft confidence map and derive a complementary background gate, as defined in Equation 15.

(15) $$G^{\left(i\right)}=1-\sigma (\varphi (H^{\left(i+1\right)})),$$

where *σ*(·) denotes the Sigmoid function and *φ*(·) is a lightweight 1 × 1 projection (used to align channel dimensions and produce a gating map). *G*^(^*^i^*^)^ highlights regions that the decoder tends to treat as background, where boundary leakage is likely to occur.

We then use *G*^(^*^i^*^)^ to select boundary-relevant details from the encoder feature and fuse them with the decoder context, as defined in Equation 16.

(16) $${\hat{Z}}^{\left(i\right)}=S^{\left(i\right)}⊙G^{\left(i\right)}+H^{\left(i+1\right)},$$

where ⊙ denotes element-wise multiplication. This reverse-additive formulation encourages the module to recover fine structures from encoder features precisely in regions that are under-emphasized by the decoder, thereby strengthening foreground–background discrimination around boundaries.

Finally, we update the decoder representation via residual refinement with upsampling, as defined in Equation 17.

(17) $$H^{\left(i\right)}={\hat{Z}}^{\left(i\right)}+\text{Up }(H^{\left(i+1\right)}),$$

where Up(·) is an upsampling operator. This residual path propagates coarse semantics while progressively injecting boundary-enhanced details from shallower encoder stages, leading to sharper contours and improved segmentation consistency.

### 2.4 Loss function

To supervise training, we adopt a compound objective that combines a pixel-wise classification loss with an overlap-based region loss, which encourages both accurate per-pixel predictions and robust contour/area consistency. Specifically, the total loss is defined in Equation 18.

(18) L=Lbce+Ldice

Let $p_{n}\in (0,1)$ denote the predicted probability at pixel *n*, and *g_n_*∈ 0, 1 the corresponding ground-truth label. With *M* pixels in a mini-batch, the binary cross-entropy loss is defined in Equation 19.

(19) $$L\text{bce}=-\frac{1}{M}\sum n=1^{M}[g_{n}\text{log }(p_{n}+\epsilon )+(1-g_{n})\text{log }(1-p_{n}+\epsilon )],$$

where *ϵ* is a small constant added for numerical stability.

To directly optimize region overlap, we employ a differentiable Dice objective, as defined in Equation 20.

(20) $$L\text{dice}=1-\frac{2\sum n=1^{M}g_{n}p_{n}+\epsilon }{\sum_{n=1}^{M}g_{n}+\sum_{n=1}^{M}p_{n}+\epsilon }.$$

This term encourages the predicted mask to match the ground truth in terms of global overlap, which is particularly beneficial for segmentation tasks with class imbalance.

Finally, the overall training loss is obtained by summing the two components, as defined in Equation 21.

(21) L=Lbce+Ldice.

## 3 Experiment and analysis

This section evaluates our method on three datasets and reports both quantitative results and qualitative comparisons against representative segmentation networks.

### 3.1 Datasets

***PSFHS2023*.** PSFHS2023 was curated by our team and served as the benchmark for the MICCAI 2023 challenge (https://ps-fh-aop-2023.grand-challenge.org/). It contains transperineal intrapartum ultrasound scans from 110 parturients (with diverse ages), totaling 1, 175 images acquired at the onset of the second stage of labor to capture fetal head station. Data were collected at three clinical sites: Nanfang Hospital, Zhujiang Hospital, and the First Affiliated Hospital of Jinan University.

***FHUandHC18*.** To further validate generalization, we additionally consider two fetal-head-only datasets: an in-house fetal head ultrasound dataset (FHU) and the public HC18 challenge dataset van den Heuvel et al. (2018). Unlike PSFHS2023, both FHU and HC18 provide annotations for the FH region only, without labeling the PS. The FHU dataset is split into a training set and a test set: the training portion includes 3, 091 images from 1, 781 pregnant women, while the test portion contains 300 images from 180 pregnant women collected across six countries. HC18 comprises 551 subjects, with 999 images for training and 355 images for testing. To probe cross-domain robustness, we also use the HC18 training set as an additional external test set when training on FHU.

### 3.2 Implementation details

All experiments were implemented in Python using PyTorch and conducted on a single NVIDIA RTX A6000 GPU. All ultrasound images and corresponding masks were resized to 256×256 before training and inference. Pixel intensities were normalized to a fixed range, and the same preprocessing and input normalization strategy was applied to all compared methods for fair comparison. The proposed model used Res2Net-50 as the CNN backbone, initialized with ImageNet-pretrained weights. The CNN-stylized Transformer branch used four attention heads, a Transformer depth of 1 for each branch, a head dimension of 64, an MLP hidden dimension of 256, and a dropout rate of 0.1. The multi-scale patch settings were set to 4, 8, 16, and 32, respectively.

The networks were optimized using Adam with an initial learning rate of 1×10^−4^. The training objective was the sum of binary cross-entropy loss and Dice loss. The batch size was set to 8, and the maximum number of training epochs was set to 300. Early stopping was adopted to reduce overfitting, and training was terminated if the validation loss did not improve for 30 consecutive epochs. During testing, the model prediction was resized back to the original evaluation resolution when necessary, and all segmentation metrics were calculated using the same evaluation scripts for all methods. For reproducibility, the random seed was fixed to 100 in all experiments.

### 3.3 Evaluation metrics

Model performance was assessed using four standard segmentation metrics: Dice coefficient (DC), volume overlap error (VOE), average symmetric surface distance (ASSD), and Hausdorff distance (HD), which jointly reflect region overlap and boundary accuracy.

We compared our method with ten representative baselines, including five CNN-centric models-UNet Ronneberger et al. (2015), Attention UNet Schlemper et al. (2019), ACCUNet Ibtehaz and Kihara (2023), MTANet Ling et al. (2023), and Rolling-UNet Liu et al. (2024)-and five Transformer-based approaches SwinUNet Cao et al. (2022), CTO Lin et al. (2023b), H2Former He et al. (2023), ScribFormer Li et al. (2024b), and DBRN Chen et al. (2024b). To ensure fairness, identical preprocessing and input normalization procedures were applied to all competing methods.

For each dataset, all quantitative metrics were computed at the image level and then averaged over the corresponding test set. Since all compared methods were evaluated on the same test images, paired statistical analysis was performed between the proposed method and each baseline. Specifically, we used a two-sided Wilcoxon signed-rank test to assess whether the improvement of the proposed method was statistically significant. A value of p*<*0.05 was considered statistically significant. We also examined the standard deviations of the main metrics across test samples, and all values were within a reasonable statistical range, indicating that the reported mean values were stable and representative. To avoid overcrowding the comparison tables, we report the mean values in the main tables and use “*” to denote statistically significant differences based on paired image-level testing.

### 3.4 Comparison on the PSFHS2023 dataset

### 3.5 Quantitative comparison

We quantitatively assess the proposed IU segmentation approach on PSFHS2023 and summarize the comparison with representative baselines in **Table 1**. Our method achieves a mean Dice score of 0.911, together with HD of 3.401, ASSD of 0.686, and VOE of 0.177. Overall, these results demonstrate clear advantages over both CNN-based and Transformer-based competitors, with statistically significant improvements (*p <* 0.05).

**Table 1:** Performance comparison of intrapartum ultrasound image segmentation with existing methods on the PSHF2023 dataset.

Methods	FH				PS				Mean				FH-PS	Methods
	DC	HD	ASSD	VOE	DC	HD	ASSD	VOE	DC	HD	ASSD	VOE	AoP	Param(M)
Model based on CNN														
UNet	0.921	4.119	1.318	0.142	0.840	3.390	0.487	0.265	0.881	3.754	0.902	0.204	7.344	22.72
AttUNet	0.923	4.105	1.311	0.142	0.862	3.371	0.478	0.234	0.892	3.738	0.894	0.188	7.423	57.16
ACCUNet	0.920	4.541	1.853	0.155	0.838	3.478	0.414	0.275	0.879	4.009	1.134	0.215	7.228	59.20
MTANet	0.935	3.490	1.242	0.122	0.863	3.351	0.383	0.241	0.899	3.420	0.812	0.181	6.515	29.71
Rolling-UNet	0.936	3.631	1.111	0.112	0.851	3.381	0.335	0.250	0.894	3.503	0.723	0.181	6.234	38.32
Model based on Transformer														
SwinUNet	0.934	3.690	1.245	0.119	0.868	3.345	0.401	0.232	0.901	3.517	0.823	0.175	6.351	27.18
CTO	0.926	3.653	1.233	0.134	0.852	3.376	0.372	0.251	0.889	3.515	0.802	0.192	6.584	59.81
H2Former	0.928	3.611	1.251	0.128	0.854	3.366	0.382	0.249	0.891	3.488	0.816	0.188	6.712	33.70
ScribFormer	0.931	3.607	1.238	0.124	0.859	3.384	0.394	0.247	0.895	3.495	0.816	0.185	6.314	50.43
DBRN	0.939	3.515	1.135	0.123	0.868	3.340	0.367	**0.231**	0.903	3.427	0.751	0.177	6.184	68.29
Ours	0.942*	3.492*	1.052*	0.114*	0.880*	3.311*	0.320*	0.241*	0.911*	3.401*	0.686*	0.177*	6.173*	39.91

The best results are highlighted in bold. * indicates the significant difference (all P-values <0.05).

In particular, the boundary-related metric ASSD shows the most pronounced gain. ASSD measures the symmetric average distance between the predicted contour and the reference contour, accounting for errors in both directions; it therefore provides a faithful indicator of boundary localization quality. The improved ASSD suggests that the proposed model recovers fine boundary structures more reliably in challenging IU images.

Finally, as reported in the last row of **Table 1**, our model also exhibits a favorable accuracy–efficiency trade-off. Despite its strong performance, the parameter count remains competitive relative to other methods, indicating better parameter efficiency. To further evaluate the deployment feasibility of the proposed method, we compared the computational complexity in terms of trainable parameters, number of floating-point operations (FLOPs), and inference time. All models were analyzed using a single 256×256 input image (batch size 1). All methods employed the same MAC-based performance analysis convention to compute FLOPs. In this setting, UNet requires approximately 46.0G FLOPs, while the proposed model requires approximately 10.50G FLOPs. Despite having more parameters than UNet, the proposed model requires significantly fewer FLOPs while achieving higher segmentation accuracy and better boundary localization performance. This is because the CNN-style Transformer branch performs global context modeling on down-resolution feature maps, thus avoiding the high computational burden of dense high-resolution convolutional decoding. Furthermore, the complete AoP estimation pipeline takes approximately 0.6 seconds on an NVIDIA Jetson Nano 4G edge device, indicating that the method is suitable for real-time intrapartum ultrasound analysis and immediate care deployment.

#### 3.5.1 Visual inspections

**Figure 5:**
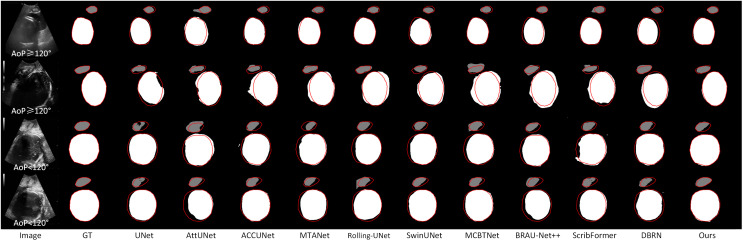
Visual comparison on the PSFH2023 dataset. The red line represents the contour of the ground truth.

**Figure 5** presents representative qualitative comparisons across different methods. For clearer clinical interpretation, we include cases with AoP *<* 120° and AoP ≥ 120°, which correspond to scenarios where cesarean delivery and vaginal delivery are typically considered, respectively. Compared with other state-of-the-art approaches, our method produces contours that adhere more closely to the reference annotations, especially around challenging boundary regions. Many competing methods exhibit a consistent bias: they often overestimate the FH area while missing parts of the PS, leading to noticeable boundary shifts relative to the ground truth. In contrast, the proposed model better preserves fine structural details and yields more accurate delineations for both FH and PS, resulting in visually more faithful segmentations. Overall, these examples confirm the advantage of our approach in boundary-sensitive IU segmentation.

### 3.6 Comparison on the FHU and HC18 datasets

To assess cross-dataset robustness, we train the model on our in-house FHU dataset and evaluate it both on the FHU test split and on an external set constructed from the training portion of the public HC18 dataset, which is used here as an additional test domain.

The quantitative results in **Table 2** show that our method achieves strong performance on FHU and maintains superior accuracy when transferred to HC18, outperforming the competing baselines and indicating robust generalization. Because both FHU and HC18 provide annotations for the FH only, most methods obtain relatively high scores; nevertheless, our approach consistently ranks first across all reported metrics.

**Table 2:** Comparative experiments on FHU and HC18 datasets.

Methods		FHU				HC18		
Metric	Dice	HD	ASSD	VOE	Dice	HD	ASSD	VOE
UNet	0.923	3.711	2.111	0.151	0.940	3.634	1.118	0.100
AttUNet	0.924	3.697	2.045	0.152	0.934	3.784	1.184	0.109
ACCUNet	0.928	3.311	1.758	0.142	0.942	3.617	1.105	0.105
MTANet	0.928	3.346	1.848	0.135	0.940	3.612	1.110	0.109
Rolling-UNet	0.930	3.333	1.834	0.144	0.941	3.617	1.108	0.095
SwinUNet	0.927	3.455	1.732	0.138	0.938	3.524	1.125	0.114
CTO	0.931	3.181	1.371	0.122	0.939	3.701	1.105	0.098
H2Former	0.926	3.344	1.719	0.133	0.941	3.666	1.120	0.102
ScribFormer	0.931	3.338	2.033	0.163	0.942	3.588	1.107	0.097
DBRN	0.932	3.255	1.422	0.125	0.945	3.611	1.105	0.094
Ours	0.939*	3.079*	1.268*	0.112*	0.949*	3.511*	1.099*	0.091*

The best results are highlighted in bold. * indicates the significant difference (all P-values <0.05).

**Figure 6:**
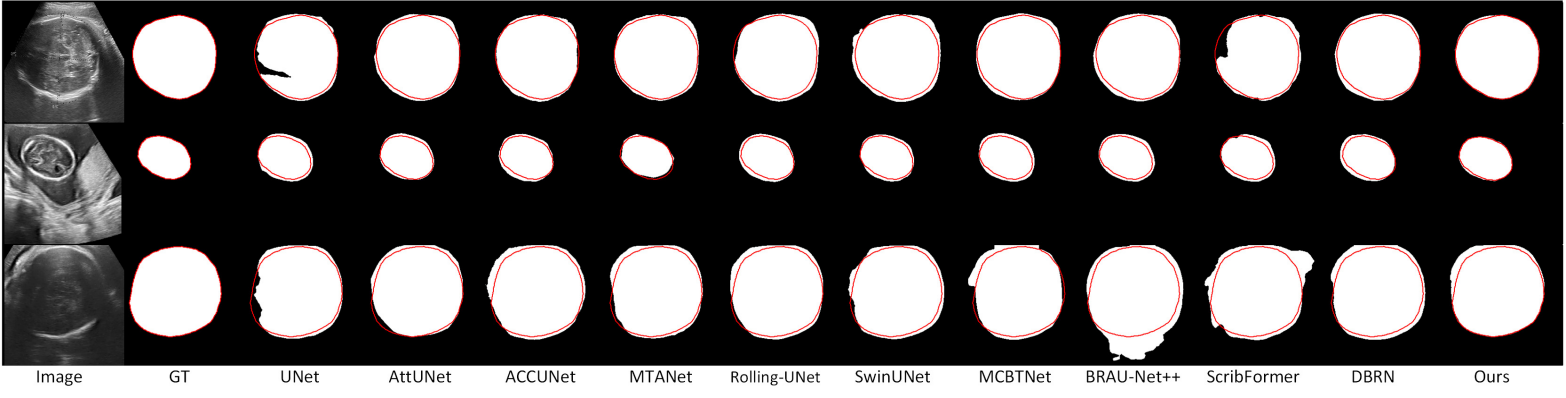
Visual comparison on the FHU and HC18 datasets. The red line represents the contour of the ground truth.

Qualitative comparisons in **Figure 6** provide additional evidence. Although several methods yield competitive numerical metrics, their predicted contours often exhibit visible boundary offsets relative to the reference masks. By contrast, our method produces cleaner and more accurate boundaries, especially in challenging transition regions. Collectively, these results demonstrate that the proposed model generalizes well across datasets and is particularly effective at recovering fine boundary structures.

### 3.7 Ablation study

We perform ablation experiments on the PSFHS2023 dataset to isolate the contribution of the major design choices in our framework.

#### 3.7.1 Effect of the CNN-stylized Transformer branch

To examine the role of the CNN-stylized Transformer branch, we construct two reduced variants: (i) a model that removes the Transformer stream entirely, and (ii) a model that replaces the CNN-stylized Transformer with a conventional non-stylized Transformer branch. The quantitative results are summarized in **Table 3**. Removing the Transformer stream leads to a clear performance drop, with the mean Dice decreasing to 0.898, indicating that long-range contextual modeling is important for this task. Replacing our CNN stylized design with a standard Transformer also degrades performance (mean Dice = 0.901), which is consistent with the attention instability commonly observed under limited medical data. In contrast, the proposed CNN-stylized Transformer branch preserves the benefits of global dependency modeling while improving attention training stability, resulting in higher segmentation accuracy and faster convergence.

**Table 3:** Ablation experiments on the PSFH2023 dataset.

Method	FH-dice	PS-dice	Mean-dice
Ours	0.942	0.880	0.911
w/o Transformer branch	0.931	0.865	0.898
non-CNN-style Transformer branch	0.936	0.867	0.901
w/o CBFE	0.938	0.868	0.903
w/o CA in CBFE	0.941	0.873	0.907
w/o SA in CBFE	0.939	0.873	0.906
w/o BA in RABR	0.937	0.866	0.901
w/o residual in RABR	0.941	0.874	0.907

#### 3.7.2 Effectiveness of the cross-branch feature exchange block

To assess the impact of the cross-branch fusion design, we replace the CBFE block with a na¨ıve feature merging strategy (i.e., direct aggregation) and report the results in **Table 3**. Without CBFE, the mean Dice drops to 0.903, whereas the parameter count is reduced by only 0.23M, indicating that CBFE delivers a meaningful accuracy gain at negligible model-size overhead. We further ablate the two internal components of CBFE by disabling its spatial-attention (SA) and channel-attention (CA) pathways. Removing SA and CA reduces the mean Dice to 0.906 and 0.907, respectively, confirming that both pathways contribute to the effectiveness of cross-branch interaction and complementary feature integration.

#### 3.7.3 Effectiveness of the reverse-additive boundary refinement module

We also evaluate the contribution of RABR by separately removing its reverse-additive boundary-attention unit and its residual refinement pathway. As summarized in **Table 3**, eliminating the boundary-attention unit lowers the mean Dice to 0.901, while disabling the residual refinement decreases the mean Dice to 0.907. These degradations suggest that RABR improves segmentation primarily by (i) explicitly mining boundary-relevant cues that are often suppressed in background responses and (ii) progressively sharpening ambiguous transitions through residual, stage-wise refinement, leading to more accurate boundary delineation.

### 3.8 Clinical manifestations

To examine clinical utility beyond segmentation accuracy, we introduce an AoP measurement error index, denoted as ΔAoP, to quantify how closely the automatically computed AoP matches the reference annotation, as defined in Equation 22.

(22) $$\text{ΔAoP}=|{\text{AoP}}_{\text{pred}}-{\text{AoP}}_{\text{gt}}|,$$

where AoP_pred_ is the AoP derived from the predicted segmentation and AoP_gt_ is the ground-truth AoP. We report the mean ΔAoP as an accuracy summary; smaller values indicate more precise AoP estimation. The results are listed in the last column of **Table 1**, showing that our method yields AoP measurements that are consistently closer to the reference values than competing approaches.

Finally, we demonstrate practical deployability by running the model on an NVIDIA Jetson Nano 4G edge device, enabling portable, point-of-care AoP assessment. Although the proposed method achieved promising segmentation and AoP estimation performance and has been preliminarily introduced into a hospital setting for trial use, these results should be interpreted as evidence of technical feasibility and potential clinical translation rather than confirmation of routine clinical deployment. The current in-hospital trial use provided preliminary clinician feedback and demonstrated the feasibility of real-time AoP estimation in a practical environment. However, it was not a formal prospective multi-center clinical validation study with predefined clinical endpoints. Therefore, the proposed method should currently be regarded as a clinician-in-the-loop assistance tool. Further prospective clinical studies are required to evaluate its robustness across different ultrasound devices, operators, and clinical environments, as well as its effects on workflow efficiency, measurement reproducibility, clinical decision-making, and maternal-neonatal outcomes.

## 4 Discussion

The proposed framework carries direct clinical relevance. Reliable estimation of fetal head position during labor can support delivery planning, help determine the likelihood of successful vaginal birth, and facilitate early recognition of malposition. By enabling more objective assessment, such tools may reduce the incidence of adverse events associated with prolonged or obstructed labor and fetal compromise. Moreover, accurate and timely positioning information can streamline intrapartum management, decrease avoidable interventions, and improve communication and confidence among both clinicians and parturients, ultimately contributing to safer delivery and smoother postpartum recovery.

In practice, intrapartum ultrasound is frequently performed under time pressure, and image acquisition may be conducted not only by obstetricians but also by nursing staff with heterogeneous training. This variability often leads to inconsistent image quality and unstable AoP measurements, increasing workload and prolonging decision-making. The situation is further complicated by uterine contractions and maternal–fetal motion, which can degrade image clarity and alter the relative spatial configuration of the fetal head and PS, yielding substantial anatomical variability across frames. Given the subjective nature of manual interpretation and the expertise required to obtain reproducible measurements, an automated AoP pipeline that is both accurate and efficient is highly desirable for routine labor monitoring.

Using the PSFHS2023 cohort, we further examined the relationship between AoP and fetal head descent. In our experiments, the proposed method achieved an average absolute AoP error of 6.173°, which is well below the commonly accepted clinical tolerance of 15°, indicating that the automated measurements are sufficiently precise for practical use.

To facilitate real-world deployment, we implemented the full pipeline on an NVIDIA Jetson Nano 4G edge platform, enabling point-of-care inference without reliance on high-end hardware. The benefit was particularly evident for newly recruited personnel, who were able to obtain accurate AoP estimates with minimal prior experience, reducing the dependence on extensive training.

From a clinical deployment perspective, uncertainty or confidence estimation is also important for improving the safety of automated AoP assessment. The current model produces deterministic segmentation outputs, whereas difficult cases such as severe acoustic shadowing, poor image quality, incomplete visualization of the fetal head or pubic symphysis, and unusual fetal head positions may require additional reliability assessment.

Despite these encouraging results, several limitations remain. The current study focuses on static 2D ultrasound images and does not explicitly model temporal dynamics in ultrasound video sequences. However, intrapartum ultrasound is a dynamic imaging process: fetal head descent, uterine contractions, maternal–fetal motion, probe pressure, and probe orientation may all change continuously during acquisition. As a result, a single frame may suffer from transient acoustic shadowing, motion blur, incomplete visualization of the fetal head or pubic symphysis, or temporary anatomical deformation. Without temporal modeling, the proposed method cannot exploit adjacent-frame continuity to enhance segmentation consistency, suppress frame-level noise, recover temporarily obscured boundary information, or characterize the dynamic trajectory of fetal head descent. This may limit the robustness of AoP estimation in challenging real-time labor-monitoring scenarios. In future work, we plan to collect and annotate intrapartum ultrasound video sequences and incorporate temporal modeling strategies, such as recurrent feature aggregation, temporal attention, optical-flow-guided feature propagation, and video-level consistency constraints, to further improve segmentation stability and AoP estimation robustness under motion and contraction-induced variability.

## 5 Conclusion

This work presents a dual-path hybrid framework for intrapartum ultrasound segmentation and automated AoP estimation. The proposed design addresses two key challenges in data-limited IU analysis—attention instability and boundary ambiguity—by combining a parallel CNN/Transformer architecture with a CBFE module for effective cross-stream fusion and a RABR module for progressive boundary enhancement. Extensive experiments on three datasets demonstrate consistent improvements over representative state-of-the-art baselines, and the resulting AoP measurements show potential for practical, point-of-care labor assessment.

## Data Availability

The datasets presented in this study can be found in online repositories. The names of the repository/repositories and accession number(s) can be found in the article/supplementary material.
